# Assessing the social climate of physical (in)activity in Canada

**DOI:** 10.1186/s12889-018-6166-2

**Published:** 2018-11-27

**Authors:** Lira Yun, Leigh Vanderloo, Tanya R. Berry, Amy E. Latimer-Cheung, Norman O’Reilly, Ryan E. Rhodes, John C. Spence, Mark S. Tremblay, Guy Faulkner

**Affiliations:** 1 School of Kinesiology, University of British Columbia, Lower Mall Research Station 337, 2259 Lower Mall, Vancouver, British Columbia V6T 1Z4 Canada; 2 ParticipACTION, Toronto, ON Canada; 3 Faculty of Kinesiology, Sport, and Recreation, University of Alberta, Edmonton, AB Canada; 4 School of Kinesiology and Health Studies, Queens University, Kingston, ON Canada; 5 College of Business and Economics, University of Guelph, Guelph, ON Canada; 6 School of Exercise Science, University of Victoria, Victoria, BC Canada; 7 Healthy Active Living and Obesity Research Group, Children’s Hospital of Eastern Ontario Research Institute, Ottawa, ON Canada

**Keywords:** Social climate, Physical activity, Policy, Public opinion, Ecological model

## Abstract

**Background:**

Ecological models suggest that a strategy for increasing physical activity participation within a population is to reconstruct the “social climate”. This can be accomplished through 1) changing norms and beliefs, 2) providing direct support for modifying environments, and 3) implementing policies to encourage physical activity. Nevertheless, surveillance efforts have paid limited attention to empirical assessment of social climate. This study responds to this gap by assessing the social climate of physical activity in Canada.

**Methods:**

A representative sample of Canadian adults (*n* = 2519, male/female = 50.3%/49.7%, *M*_*a*ge_ = 49.1 ± 16.3 years) completed an online survey asking them to assess social climate dimensions including social norms of physical (in)activity, perceptions of who causes physical inactivity and who is responsible for solving physical inactivity, and support for physical activity-related policy. Descriptive statistics (frequencies) were calculated. Multinomial logistic regressions were constructed to identify whether demographic variables and physical activity participation associated with social climate dimensions.

**Results:**

Physical inactivity was considered a serious public health concern by 55% of the respondents; similar to unhealthy diets (58%) and tobacco use (57%). Thirty-nine percent of the respondents reported that they often see other people exercising. Twenty-eight percent of the sample believed that society disapproves of physical inactivity. The majority of respondents (63%) viewed the cause of physical inactivity as both an individual responsibility and other factors beyond an individuals’ control. Sixty-seven percent of respondents reported physical inactivity as being both a private matter and a public health matter. Strong support existed for environmental-, individual-, and economic-level policies but much less for legislative approaches. The social climate indicators were associated with respondents’ level of physical activity participation and demographic variables in expected directions.

**Conclusion:**

This study is the first known attempt to assess social climate at a national level, addressing an important gap in knowledge related to advocating for, and implementing population-level physical activity interventions. Future tracking will be needed to identify any temporal (in)stability of these constructs over time and to explore the relationship between physical activity participation and indicators of the national social climate of physical activity.

## Background

Physical inactivity is an important cause of chronic disease and is a major public health concern in Canada. Nevertheless, the prevalence of physical inactivity is growing [1]. According to the Canadian Health Measures Survey (CHMS; [2]), objectively measured physical activity data demonstrated just over 2 in 10 adults and 1 in 10 children and youth met the Canadian physical activity guidelines (www.csep.ca/guidelines). A holistic approach targeting structural and systemic change is recommended for reversing declines in physical activity and to influence behavior of an entire population [3]. Ecological models provide a framework that can guide the development of a comprehensive intervention targeting systematic mechanisms of change at each level of determinants from individual, social, environmental, to policy levels [4, 5].

Environments are multidimensional and can be described as social or physical, built or natural, actual or perceived, and can include constructs such as social climate – a psychological term that refers to the general feelings, attitudes, beliefs and opinions on a subject within society [6]. Thus, a means to achieve behavior change of whole populations may be to de-normalize physical inactivity and re-normalize physical activity through *changing norms and beliefs* and by providing *direct support* for modifying environments and policies to encourage physical activity [4]. Further, individuals’ unhealthy behaviors and lifestyles may be modified through the alteration of social climate [6, 7]. For instance, de-normalizing smoking has been one of the most effective strategies in reducing smoking prevalence through policies aimed at restricting smoking in public in combination with health advocacy and media efforts to shift the social acceptability of smoking [8–11]. Programs and policy actions that reinforced the message that tobacco use is *not* a normal activity have changed public perceptions of the social acceptability of smoking [12]. In turn, this decreased social acceptability of smoking has been shown to reduce tobacco use as well as build public support of tobacco control regulations. However, whether there is a comparable role for the social climate regarding physical (in)activity to influence policy enactment and population changes in behavior remains to be seen.

Based on a number of theoretical frameworks influencing health-related behaviors [13, 14] and a set of related studies exploring aspects of social climate related to tobacco control [15] and obesity [16, 17], a range of potential factors reflecting social climate can be identified, including: 1) the social norms and acceptability of physical (in)activity, 2) perceptions of the causes of physical inactivity, 3) perceptions of responsibility for preventing physical inactivity, and 4) the social acceptability of different policies and regulations in addressing physical inactivity.

As noted, social norms – a pattern of behaviors or beliefs generally held by ‘society’ – are one salient construct within a broader conceptualization of social climate. Conceptually, how serious a health issue is recognized in one’s society (c.f., Health Belief Model [18], Protection Motivation Theory [19]) is theorized to guide one’s behavior. Perceived seriousness of physical inactivity, compared to other public health issues, may reflect the degree and magnitude of social beliefs regarding physical activity as a public health concern [20]. Perceptions of how common it is to see people exercising in their environment (*descriptive norms*) and perceptions of whether others approve or disapprove of physical (in)activity (*subjective or injunctive norms*) are also proposed to reflect the social climate of physical (in)activity [21, 22]. Previous research applying social cognitive theories have posited that individuals are more likely to be physically active if they perceive that the social expectations are to be active [23]. However, it is important to note that social norms are commonly found to be weak predictors of physical activity behavior compared to risk behaviors such as drinking, smoking, and drug use [24–26]. Although speculative, this may be due to the potential harm to others of engaging in risky behaviors (e.g., drinking and driving, secondhand smoking) whereas physical (in)activity may not directly affect others’ health.

The social ecological approach further acknowledges that collective norms reflect one’s perceptions of political actors, social institutions, and causes of social issues [7]. Internal or external attributions of a public health issue (in this case, physical inactivity) and perceptions of individual or societal responsibility for solutions are linked to expectations of societal actions and acceptance of policies to address the issue [27]. Previous studies have proposed that societal solution attributions are positively associated with support for public policies [28]. People who hold internal causal attributions for health are more likely to perceive themselves responsible whereas those who hold external causal attributions of health problems are more likely to support societal actions including policy, legislation, and regulation [29, 30]. Public beliefs about what causes physical inactivity as well as who is responsible for addressing physical inactivity likely reflect the physical activity social climate at a population level.

A final component of social climate measured in the current study is public support for different policy solutions for physical inactivity. Here, policy interventions vary in their level of intrusiveness [31]. Policies targeting individual responsibility for behaviors (e.g., media campaigns promoting the benefits of physical activity and/or harms of physical inactivity) are less intrusive and less forceful while modifying community environments (e.g., quantity and quality of green spaces, safe areas for physical activity, and the design of neighborhoods to encourage informal physical activity) or economic level supports (e.g., providing incentives, subsidies and tax credits around physical activity) are more intrusive. Public resistance to policy may cause problems with implementation and adherence, and ultimately result in its withdrawal from consideration [32]. Conversely, public support for different policy interventions may be a prerequisite for policy makers in developing and implementing those policies. Social climate may therefore indirectly influence population level physical activity through the implementation of more effective policy approaches that have public support.

In other health domains, research has included monitoring beliefs about smoking, perceived harm, social influences, and attitudes to regulations (e.g., International Tobacco Control Policy Evaluation Project [33]). This work provided guidance in the development and implementation of policies to reduce tobacco use. Similarly, the body of research assessing aspects of social climate with regard to obesity has grown in the last decade. For example, Raine et al. [17] examined perceptions among Canadian ‘policy influencers’ of the causes of, responsibility for, and levels of support for different policies addressing obesity. They found that most policy influencers viewed all risk behaviors as personal responsibilities (47.0% for alcohol, 55.5% for obesity, 59.3% for tobacco, 69.1% for physical activity, and 63.1% for healthy eating), while one-fifth (for healthy eating and physical activity) to one-third (for obesity, alcohol, and tobacco) viewed the responsibility to be both personal and societal. Most policy influencers indicated their organization has at least some responsibility for obesity prevention programs and policies (i.e., approximately 70% for encouraging healthy eating and 90% for physical activity). Policies with very strong overall support were those aimed at individual responsibility for behaviors, such as providing programs to educate the general public and implementing programs aimed at changing school environments. However, policies to change the design of workplaces and communities to encourage informal physical activity and those that affect economic measures and legislations received weak overall support [17].

Similar results have also been reported among the general public in the UK, USA, and Germany such that being overweight or obese was mainly perceived to be an individual’s own fault [34–36]. Furthermore, less intrusive policies targeting individuals through school-based programs and campaigns are generally more supported than more intrusive actions such as restricting unhealthy foods at restaurants and increasing taxes on the sale of unhealthy foods. Most recently, both public (38.7%) and health professionals (32.2%) in Canada viewed obesity as a community problem of bad food and inactivity followed by a personal problem stemming from bad choices (31.7% for public and 28.7% for health-care professionals [37]). Bhawra et al. [38] reported that Canadian youth and young adults strongly supported menu labelling in restaurants and schools, and food package symbols whereas taxation, zoning restrictions, and bans on marketing to children received relatively lower levels of support.

Past empirical measurement and analysis of social climate has paid less attention to the context of physical (in)activity. We are not aware of studies directly assessing the social climate of physical (in)activity beyond the context of obesity. Rather, previous research has largely applied social cognitive models focused on normative beliefs and not a broader conceptualization of social norms. This study seeks to address this gap. As social climate change is often central to public health policy agendas, the purpose of this study was to assess the social climate of physical (in)activity in Canada and to examine whether the social climate dimensions were related to individuals’ behavior. Such an improved understanding will allow for the identification of a benchmark that can be reassessed over time to determine if social climate changes in response to broader policy and programmatic initiatives, or whether changes in the social climate precede consideration and implementation of greater policy and legislative innovation to facilitate a more active Canada.

## Methods

### Sample and recruitment

A total of 2519 participants were recruited from a representative sample of panelists (Canadian adults ≥18 years) drawn from the Angus Reid Forum. This forum includes 100,000 Canadians who have already consented to participating in survey research before joining the panel. The panel is comparable with the Canadian census in terms of age, sex, region, income, employment, and language spoken. Panelists generally receive a small cash reward ($0.50–$3 CAD), after completing a survey. By enrolling as a panelist in the Angus Reid Forum, recruited individuals consented to their participation in invited surveys or panel discussions. Ethical approval was not needed according to article 2.4 and 5.5 of the Tri-Council policy statement (TCPS2) regarding ethical conduct of human research reporting on secondary analyses of minimal risk and anonymous data [39]. The survey was conducted by PArticipACTION, a Canadian non-profit organization promoting physical activity (www.participaction.com) as part of its ongoing public relations and advocacy work.

### Measures

#### Survey development procedure

The survey instrument was initially generated drawing on similar questions that have been used to assess the social climate regarding obesity and tobacco control [17, 40], including the International Tobacco Control Survey (http://www.itcproject.org). The questionnaire aimed to solicit information regarding the social climate of physical activity in Canada including the perceived seriousness of physical (in)activity as a public health issue, social norms of physical (in)activity, attributions of causes and responsibility in solving physical inactivity, and level of support for physical activity related policies, regulations and programs. Demographics and physical activity participation were also assessed. Once drafted, the questionnaire was distributed to members of two advisory groups of ParticipACTION to solicit feedback and help enhance face and content validity [41]. The questionnaire was piloted online (*n* = 35) to ensure the questions and language were clear and comprehensible. Test-retest reliability was examined by performing intraclass correlations (ICC) using a two-way random effects model, absolute agreement with a sample of 100 participants. Moderate reliability [42] across all (ICC scores ≥ .70) except for one item (society disapproves of physical inactivity, ICC = .33, 95% CI: 0.02, 0.38) was demonstrated. The final survey is available upon request to the first author.

#### Seriousness of public health issues

The first section of the survey covered perceived seriousness of different health risk behaviors based on a similar question examining public opinion of obesity [16]. Health issues included physical inactivity, sitting too much (sedentary behavior), tobacco use, alcohol misuse, cannabis use, unhealthy diets, and lack of sleep. Respondents were asked to rate perceived seriousness on a 7-point scale ranging from “not at all serious (1)” to “very serious (7).”

#### Social norms

Modified from a previous study [43], three descriptive norm items were used as follows: “I often see other people walking in my neighborhood”, “I often see other people exercising (e.g., jogging, bicycling, playing sports) in my neighborhood”, and “I often see kids playing actively outdoors (e.g., playing games like tag, sports, riding their bikes) in my neighborhood”. Response options for each item were on a 7-point scale ranging from “strongly disagree (1)” to “strongly agree (7)”. Additionally, respondents were asked to estimate the proportion of Canadians their age meeting physical activity guidelines on a scale ranging from “0%” to “100%” within a ten-percentage interval. One item measured injunctive norms of physical activity: “How many people who are important to you (e.g., friends or family) would you say engage in 150 minutes of moderate-to-vigorous physical activity per week?” This item was rated on a 5-point scale ranging from “all of them” to “none of them.” The acceptability of physical inactivity was also measured using one item: “Society disapproves of physical inactivity.” Responses on the item were made on a 7-point scale ranging from “strongly disagree (1)” to “strongly agree (7)”. All items had the following instruction: “Please indicate how much you agree or disagree with the following statements. In considering these questions, think about where you live during the spring or fall seasons.”

#### Perceptions of the causes and responsibility in solving physical inactivity

Items were based on questions used to assess perceptions of obesity [17, 36]. Perceptions of the causes of inactivity were assessed on one of the following options of physical inactivity as “an individual’s fault”, “caused by other factors beyond an individual’s control”, “both an individual’s fault and caused by other factors beyond an individual’s control”, “neither an individual’s fault nor caused by other factors beyond an individual’s control”, and “don’t know”. Responsibility to solve the issue of physical inactivity was measured on one of the following options of physical inactivity as “a private matter that people need to deal with on their own”, “a public health matter that society needs to solve”, “both a private matter and a public health matter”, “neither a private matter nor a public health matter”, and “don’t know.”

#### Support for physical activity-related policy

Support for physical activity-related policies, regulations and programs was assessed to address the following: 1) individual responsibility for behaviors (e.g., providing programs to educate or motivate the general public about the importance of regular physical activity), 2) modifying community environments (e.g., the quantity and quality of green spaces, safe areas for physical activity, and the design of neighborhoods to encourage informal physical activity), 3) targeting legislative changes to modify the environment (e.g., banning all traffic in high-use pedestrian areas during peak hours to support active or public transportation and restricting the use of elevators for trips to lower floors), 4) focusing on economic levers (e.g., incentives, subsidies, and tax credits). Each of the items was measured on a 7-point scale ranging from “strongly oppose (1)” to “strongly support (7)”. Items were initially generated using those reported by Raine et al. [17] and supplemented with five items generated through feedback from the consultations with the advisory groups.

#### Physical activity participation

The Physical Activity for Adults Questionnaire (PAAQ) was administered to measure respondents’ physical activity participation [44]. The PAAQ captures total time spent doing moderate to vigorous-intensity physical activity (MVPA) in three domains: transportation, leisure time, and ‘other’ including work, home and volunteering. Respondents were asked to report activities that lasted at least 10 consecutive minutes. Once completed, a total amount of MVPA in the last 7 days was calculated. Previous research has supported the validity of the PAAQ [44]).

#### Demographics

Respondents reported sex, age, dwelling (urban, semi-urban, rural), and household income level (in increments from <$35,000 to > $125,000, don’t want to report).

### Data collection procedures

The survey was deployed online in French and English on January 15, 2018 and remained open for 7 days. Participants were sent an email with a link to the survey, with a follow-up reminder email being re-sent 2 days before the survey closed. The survey required approximately 15–20 min to complete. Once closed, all data were cleaned, de-identified, and tabulated into an SPSS (IBM, New York, USA).

### Data analyses procedures

Descriptive statistics are reported as frequencies and percentages. Multinomial logistic regressions were constructed to assess the associations of sociodemographic factors with social climate dimensions. Sex (female, male), age, household income level (< $35,000, ≤$35,000 to <$75,000, ≤$75,000 to <$125,000, and $125,000 ≤), dwelling (urban, semi-urban, rural), and meeting the Canadian physical activity guidelines (achieving 150mins/week MVPA or not) served as predictors. For ease of interpretation, responses to the Likert scales for the seriousness of physical inactivity, response of social norms of physical activity and policy support questions were collapsed and recoded to three-level outcome variables (e.g., “strongly and moderately disagree” vs. “slightly disagree, neutral, and slightly agree” vs. “moderately and strongly agree”). This coding method was modified from a previous study [45]. Perceptions of the causes of physical inactivity were recoded to “individual cause”, “societal cause”, and “both individual and societal cause”. Perceptions of responsibility for solving physical inactivity were recoded to “private matter”, “public health matter”, and “both private and public health matter”. A linear regression was conducted to assess the relationship between socio-demographic factors and the perception of the proportion of Canadians their age meeting the guidelines. Statistical analyses were conducted using SPSS version 24 and all statistical inferences were based on an alpha of *p* < 0.01.

## Results

The sociodemographic characteristics of the respondents are described in Table 1. The sample was well distributed in terms of sex (male = 50.3%), age (*mean* = 49.06 ± 16.28 years, range 18–92), income (< $35,000 = 16.2%, ≤ $35,000 to <$75,000 = 28.5%, ≤ $75,000 to < $125,000 = 25%, > $125,000 = 13.1%, no response = 17.1%), and dwelling setting (urban = 54.1%, semi-urban = 26.5%, rural = 19.4%). Among the total sample of the respondents, only 404 (16.0%) reported engaging in a minimum of 150 min of MVPA per week and were categorized as the ‘active’ group.

**Table 1:** Sociodemographic characteristics of participants (*N* = 2519)

	Frequency	Percentage
Sex		
Female	1253	49.7
Male	1266	50.3
Age group (years)		
18–29	356	14.1
30–39	468	18.6
40–49	432	17.1
50–59	516	20.5
60–69	425	16.9
70 and above	322	12.8
Income level (Canadian dollars)		
< $35,000	407	16.2
$35,000 to <$75,000	719	28.5
$75,000 to <$125,000	630	25.0
$125,000<	331	13.1
Don’t want to report	432	17.1
Dwelling setting		
Urban	1363	54.1
Semi urban	668	26.5
Rural	488	19.4
Active group		
Inactive	1822	72.3
Active	404	16.0
Total	2226	88.4
Missing	293	11.6

The respondents rated “not enough physical activity” (55.1% reported very or moderately serious) as serious as “unhealthy diets” (57.7%) and “tobacco use” (56.8%). “Alcohol misuse” (50.3%) was also rated fairly serious followed by “lack of sleep” (41.4%) and “sitting too much” (38.7%). “Cannabis use” was rated the least serious issue (33.3%).

Sex, income, and active group membership were associated with perceived seriousness of physical inactivity (see Table 2). Specifically, respondents who were female were more likely to report greater seriousness (vs. not serious) and individuals of higher income and who were physically active were more likely to report greater seriousness (vs. neutral) of physical inactivity compared to their counterparts.

**Table 2:** Likelihood of support for perceptions of seriousness and social norms of physical (in)activity

	Seriousness of physical (in)activity	
	B (SE)	OR (95% CI)
Not serious^b^		
Intercept	−1.16(0.83)	
Age	−0.02 (.01)	0.98 (0.96–1.00)
Income	−0.36 (0.17)	0.70 (0.50–0.99)
Sex (female)	−1.39 (0.39)	0.25 (0.12–0.54)^**^
Sex (male)	Reference	
Dwelling (urban)	− 0.43 (0.47)	0.65 (0.26–1.63)
Dwelling (semi-urban)	0.45 (0.47)	1.57(0.63–3.92)
Dwelling (rural)	Reference	
PA^a^ (inactive)	0.49 (0.46)	1.63 (0.66–3.98)
PA^a^ (active)	Reference	
Neutral^b^		
Intercept	0.09 (0.25)	
Age	−0.001 (0.003)	1.00 (0.99–1.00)
Income	−0.18 (0.05)	0.83 (0.75–0.92)^**^
Sex (female)	−0.22 (0.10)	0.80 (0.66–0.97)
Sex (male)	Reference	
Dwelling (urban)	−0.08 (0.13)	0.93 (0.72–1.19)
Dwelling (semi-urban)	−0.12 (0.14)	0.89 (0.67–1.18)
Dwelling (rural)	Reference	
PA^a^ (inactive)	0.42 (0.13)	1.53 (1.19–1.96)^*^
PA^a^ (active)	Reference	
*R*^2^ (Nagelkerke)	.035	

^*^ *p* < .01, ^**^ *p* < .001

^a^Those who do (vs. do not) achieve 150 min of moderate-to-vigorous physical activity a week

^b^The reference category is: serious

The majority of the sample agreed that they often see other people walking in the neighborhood (*N* = 1406, 55.8%), while less than half of the respondents agreed that they often see other people exercising in the neighborhood (*N* = 989, 39.3%). Only a quarter of the sample agreed that they often see children playing actively outdoors in the neighborhood (*N* = 647, 25.6%). In general, older adults, those living in urban or semi-urban settings, and individuals meeting physical activity guidelines had higher odds of reporting seeing people walking or exercising, or kids playing actively outdoors in their neighborhood (see Table 3).

**Table 3:** Likelihood of support for social norms of physical (in)activity

		Descriptive norms						Society disapproves of inactivity		Injunctive norms of physical activity		
		Walking		Exercising		Kids active						
		B (SE)	OR (CI)	B (SE)	OR (CI)	B (SE)	OR (CI)	B (SE)	OR (CI)		B (SE)	OR (CI)
Disagree^b^										None^c^		
	Intercept	0.11 (0.47)		0.22 (0.40)		0.12 (0.34)		−0.00 (0.37)			−1.11 (0.57)	
	Age	−0.02 (0.01)	0.99 (0.97–1.00)	− 0.01 (0.01)	0.99 (0.98–1.00)	0.003 (0.004)	1.00 (1.00–1.01)	− 0.001 (0.00)	0.10 (0.99–1.01)		0.002 (0.01)	1.00 (0.99–1.02)
	Income	−0.16 (0.10)	0.19 (0.70–1.04)	− 0.16 (0.08)	0.85 (0.72–1.00)	−0.03 (0.07)	0.97 (0.85–1.11)	0.08 (0.07)	1.08 (0.93–1.25)		−0.17 (0.11)	0.84 (0.68–1.06)
	Sex (female)	− 0.41 (0.19)	0.66 (0.45–0.97)	−0.18 (0.16)	0.84 (0.61–1.15)	− 0.05 (0.13)	0.95 (0.74–1.23)	− 0.37 (0.15)	0.69 (0.52–0.91)		0.09 (0.22)	1.09 (0.71–1.68)
	Sex (male)	Reference		Reference		Reference		Reference			Reference	
	Dwelling (urban)	−1.21 (0.23)	0.30 (0.19–0.47)**	−0.85 (0.20)	0.43 (0.29–0.63)**	− 0.42 (0.17)	0.66 (0.47–0.92)	− 0.43 (0.18)	0.65 (0.46–0.92)		− 0.24 (0.28)	0.78 (0.45–1.36)
	Dwelling (semi-urban)	−0.76 (0.25)	0.47 (0.29–0.76)*	− 0.70 (0.23)	0.50 (0.32–0.78)*	− 0.59 (0.20)	0.55 (0.38–0.82)*	− 0.84 (0.23)	0.43 (0.28–0.67)**		0.29 (0.32)	1.34 (0.72–2.51)
	Dwelling (rural)	Reference		Reference		Reference		Reference			Reference	
	PA^a^ (inactive)	−0.18 (0.23)	0.84 (0.53–1.31)	0.03 (0.20)	1.03 (0.70–1.51)	0.14 (0.16)	1.15 (0.84–1.58)	0.07 (0.18)	1.08 (0.76–1.53)		1.03 (0.30)	2.79 (1.54–5.05)*
	PA^a^ (active)	Reference		Reference		Reference		Reference			Reference	
Neutral^b^										A few/some^c^		
	Intercept	0.53 (0.27)		0.81 (0.27)		0.95 (0.30)		1.31 (0.29)			1.04 (0.35)	
	Age	−0.02 (0.003)	0.98 (0.98–0.99)**	−0.01 (0.003)	0.99 (0.98–0.99)**	−0.01 (0.004)	0.99 (0.98–1.00)	− 0.01 (0.00)	0.99 (0.98–0.99)**		0.00 (0.00)	1.00 (0.99–1.01)
	Income	− 0.01 (0.05)	0.99 (0.89–1.10)	−0.04 (0.05)	0.97 (0.87–1.07)	− 0.01 (0.06)	0.99 (0.88–1.11)	0.02 (0.06)	1.02 (0.91–1.14)		0.05 (0.07)	1.05 (0.92–1.21)
	Sex (female)	− 0.24 (0.10)	0.79 (0.64–0.96)	−0.22 (0.10)	0.80 (0.66–0.98)	− 0.11 (0.12)	0.90 (0.72–1.12)	− 0.20 (0.11)	0.82 (0.66–1.02)		0.14 (0.14)	1.15 (0.87–1.51)
	Sex (male)	Reference		Reference		Reference		Reference			Reference	
	Dwelling (urban)	−0.30 (0.14)	0.74 (0.57–0.96)	− 0.30 (0.14)	0.74 (0.57–0.97)	− 0.30 (0.16)	0.74 (0.54–1.00)	− 0.26 (0.15)	0.77 (0.58–1.04)		− 0.08 (0.18)	0.93 (0.65–1.32)
	Dwelling (semi-urban)	− 0.21 (0.15)	0.81 (0.60–1.09)	− 0.17 (0.15)	0.84 (0.62–1.14)	− 0.30 (0.18)	0.75 (0.53–1.05)	0.10 (0.17)	1.10 (0.79–1.54)		0.24 (0.22)	1.27 (0.83–1.94)
	Dwelling (rural)	Reference		Reference		Reference		Reference			Reference	
	PA^a^ (inactive)	0.18 (0.13)	1.20 (0.92–1.56)	0.43 (0.13)	1.54 (1.19–1.99)*	0.43 (0.14)	1.54 (1.16–2.05)*	0.22 (0.14)	1.24 (0.94–1.64)		0.64 (0.16)	1.90 (1.39–2.58)**
	PA^a^ (active)	Reference		Reference		Reference		Reference			Reference	
	*R*^2^ (Nagelkerke)		.042		.033		.020		.039			.024

* *p* < .01, ** *p* < .001

^a^Those who do (vs. do not) achieve 150 min of moderate-to-vigorous physical activity a week

^b^The reference category is: agree

^c^The reference category: most people

A minority (*N* = 706, 28%) of the respondents believed that society disapproves of physical inactivity. Older respondents and those living in semi-urban settings were more likely to agree with the statement that society disapproves of physical inactivity (see Table 3). Overall, participants perceived that 35% of Canadians their age met physical activity guidelines. Participants who were younger (*β* = − 0.19, *p* < .001, η^2^ = 0.07), had lower income (*β* = − 0.08, *p* < .01, η^2^ = 0.04), or were active (*β* = 0.07, *p* < .01, η^2^ = 0.02) reported a higher percentage of Canadians their age to be meeting the guidelines.

In terms of injunctive norms, 2.5% of the respondents reported *all*, and 10.8% of the respondents (total 13.3%) reported *most*, people important to them engage in 150 min of MVPA per week (i.e., meet the guidelines). Physical activity participation was the only predictor of injunctive norms such that active participants had higher odds of reporting more people important to them engaging in physical activity compared to those in the inactive group (see Table 3).

The majority of respondents endorsed physical inactivity as both an individual and societal responsibility (*N* = 1578, 62.5%), followed by only an individual’s fault (*N* = 711, 28.2%), only societal responsibility (*N* = 129, 5.1%), don’t know (*N* = 52, 2.1%), and neither an individual nor societal responsibility (*N* = 49, 1.9%). Similarly, most respondents (*N* = 1678, 66.6%) viewed physical inactivity as both a private and public health matter, while 20.5% (*N* = 517) of the respondents responded that physical inactivity is a private matter that people need to deal with on their own. Only a small number of respondents viewed physical inactivity as a public health matter (*N* = 237, 9.4%), neither a private nor a public health matter (*N* = 29, 1.2%), and don’t know (*N* = 58, 2.3%). Respondents who were older, had higher income, or were active, showed higher odds of attributing physical inactivity to individual than to societal causes and endorsed physical inactivity more as a private matter than as a public health matter or both a private and a public health matter (see Table 4).

**Table 4:** Likelihood of support for perceptions of causes and solutions of physical inactivity

	Cause of inactivity		Solution of inactivity	
	B (SE)	OR (95% CI)	B (SE)	OR (95% CI)
Disagree^b^				
Intercept	−0.16 (0.58)		−0.16 (0.58)	
Age	−0.02 (0.01)	0.98 (0.97–1.00)	−0.02 (0.01)	0.98 (0.97–1.00)
Income	−0.48 (0.12)	0.62 (0.49–0.79)^**^	−0.48 (0.12)	0.62 (0.49–0.79)^**^
Sex (female)	−0.20 (0.24)	0.82 (0.51–1.32)	−0.20 (0.24)	0.82 (0.51–1.32)
Sex (male)	Reference		Reference	
Dwelling (urban)	0.32 (0.30)	1.38 (0.76–2.48)	0.32 (0.30)	1.38 (0.76–2.48)
Dwelling (semi-urban)	−0.30 (0.39)	0.74 (0.34–1.60)	− 0.30 (0.39)	0.74 (0.34–1.60)
Dwelling (rural)	Reference		Reference	
PA^a^ (inactive)	0.40 (0.31)	1.49 (0.82–2.71)	0.40 (0.31)	1.49 (0.82–2.71)
PA^a^ (active)	Reference		Reference	
Neutral^b^				
Intercept	0.76 (0.28)		0.76 (0.28)	
Age	−0.01 (0.00)	0.99 (0.99–1.00)	−0.01 (0.00)	0.99 (0.99–1.00)
Income	−0.17 (0.06)	0.85 (0.76–0.94)^*^	−0.17 (0.06)	0.85 (0.76–0.94)^*^
Sex (female)	0.39 (0.11)	1.48 (1.19–1.83)^**^	0.39 (0.11)	1.48 (1.19–1.83)^**^
Sex (male)	Reference		Reference	
Dwelling (urban)	0.27 (0.14)	1.31 (1.00–1.71)	0.27 (0.14)	1.31 (1.00–1.71)
Dwelling (semi-urban)	0.41 (0.16)	1.50 (1.10–2.05)	0.41 (0.16)	1.50 (1.10–2.05)
Dwelling (rural)	Reference		Reference	
PA^a^ (inactive)	0.36 (0.13)	1.44 (1.11–1.86)^*^	0.36 (0.13)	1.44 (1.11–1.86)^*^
PA^a^ (active)	Reference		Reference	
*R*^2^ (Nagelkerke)	.047		.047	

^*^ *p* < .01, ^**^ *p* < .001

^a^Those who do (vs. do not) achieve 150 min of moderate-to-vigorous physical activity a week

^b^The reference category is: agree

Table 5 describes the response rates of strong and moderate support for different policy approaches. The findings indicate overall strong support for individual-level policies, such as providing programs to educators (65.4%) and the general public (55.6%), and creating and sharing physical activity guidelines (52.6%). However, fewer respondents indicated strong or moderate support for funding media campaigns to educate the public (39.1% of total support). Policies to change community environments [i.e., improve universal accessibility (67.3%), providing more green spaces (67.1%), implementing transportation policies designed to promote physical activity through safe routes (55.8%), and changing the design of neighborhoods (55%)], as well as policies focusing on economic levers [i.e., providing incentives (63.9%), subsidies (57.7%), tax credits (56%) for physical activity participation and removing sales taxes on all physical activity equipment (56.7%)] were both well supported across all policy actions. However, legislative policies targeting community infrastructure and facilities received the weakest support such that only 14% of the respondents “strongly supported” banning all traffic in high-use pedestrian areas during peak hours and restricting the use of elevators for trips three floors or less (14%).

**Table 5:** Level of support for policy approaches to promote physical activity

Category	Policy support	Strongly support	Moderately support	Total level of support
Individual	Increase training of educators and school support staff to deliver quality physical activity programming	867	781	1648
		34.4%	31%	65.4%
	Provide programs to educate, inspire, support, or motivate the general public about the importance of regular physical activity	677	723	1400
		26.9%	28.7%	55.6%
	Create and share guidelines for adults that provide guidance on physical activity, sedentary behavior and sleep	569	756	1325
		22.6%	30%	52.6%
	Fund media campaigns to educate the public about increasing physical activity and reducing screen time	410	574	984
		16.3%	22.8%	39.1%
Environment (school/community)	Provide mandatory daily physical education or physical activity requirements in all schools	1332	568	1900
		52.9%	22.5%	75.4%
	Improve universal accessibility (e.g., wheelchair access) of recreation facilities to enable participation among all ability groups	1066	630	1696
		42.3%	25%	67.3%
	Enhance the quantity and quality of green spaces in all neighbourhoods	1011	680	1691
		40.1%	27%	67.1%
	Implement transportation policies designed to promote physical activity through safe routes, cycle facilities, adequate lighting, etc.	758	647	1405
		30.1%	25.7%	55.8%
	Change the design of our neighbourhoods and communities to encourage informal physical activity in daily life	713	672	1385
		28.3%	26.7%	55%
	Provide support to guarantee safe and supported play areas in urban environments (e.g., security/chaperone at an urban basketball court).	656	700	1356
		26%	27.8%	53.8%
Environment (legislative)	Redirect government funding for high performance sport (e.g., Olympians) to recreational sport	371	516	887
		14.7%	20.5%	35.2%
	Ban all traffic in high-use pedestrian areas during peak hours to support active (walking, cycling) or public transportation	347	424	771
		13.8%	16.8%	30.6%
	Restrict the use of elevators for trips three floors or less (e.g. exceptions include use by individuals with disabilities, persons with baby strollers)	351	414	765
		13.9%	16.4%	30.4%
Economics	Provide incentives for workplaces to develop physical activity policies and access to physical activity facilities for workers	892	717	1609
		35.4%	28.5%	63.9%
	Subsidize programs that encourage people to be physically active	762	691	1453
		30.3%	27.4%	57.7%
	Remove sales taxes on all physical activity equipment	854	575	1429
		33.9%	22.8%	56.7%
	Provide tax credits or monetary incentives for people who are involved in physical activity	839	571	1410
		33.3%	22.7%	56%

Female respondents demonstrated higher likelihood of supporting individual, community environment, and economic level policy although no difference was found in legislative policy support by sex (see Table 6). The associations between respondents’ age and the level of support varied by different policy domains such that older respondents were more likely to support individual level policy approaches, while younger respondents were more likely to support economic approaches. Income also predicted different policy actions: the lower income group was more likely to support legislative policy than the higher income group; however, no differences were seen in all other policy categories. Finally, active respondents were more likely to support individual and legislative policy, but not economic and community environment policy support.

**Table 6:** Odds of support (95% confidence interval) for perceptions of causes and solutions of physical inactivity

	Individual		Community		Economic		Legislative	
	B (SE)	OR (CI)	B (SE)	OR (CI)	B (SE)	OR (CI)	B (SE)	OR (CI)
Disagree^6^								
Intercept	−1.41 (0.71		−4.72 (1.20)		−4.80 (0.81)		−1.16 (0.42)	
Age	−0.02 (0.01)	0.98 (0.96–1.00)	− 0.003 (0.01)	1.00 (0.97–1.02)	0.03 (0.01)	1.03 (1.01–1.05)^*^	0.004 (0.01)	1.00 (0.99–1.01)
Income	0.02(0.14)	1.02 (0.77–1.34)	0.49 (0.22)	1.63 (1.07–2.48)	0.37 (0.15)	1.44 (1.07–1.94)	0.24 (0.08)	1.27 (1.07–1.49)^*^
Sex (female)	−0.51 (0.28)	0.60 (0.35–1.04)	−1.23 (0.47)	0.29 (0.12–0.74)^*^	−0.64 (0.30)	0.53 (0.30–0.95)	0.14 (0.17)	1.15 (0.83–1.59)
Sex (male)	Reference		Reference		Reference		Reference	
Dwelling (urban)	−0.53 (0.35)	0.59 (0.30–1.16)	0.77 (0.76)	2.17 (−.49–9.58)	−0.40 (0.34)	0.67 (0.34–1.32)	0.19 (0.22)	1.21 (0.78–1.86)
Dwelling (semi-urban)	−0.32 (0.39)	0.73 (0.34–1.55)	1.08 (0.79)	2.94 (0.62–13.87)	−0.24 (0.40)	0.79 (0.36–1.71)	0.18 (0.25)	1.20 (0.74–1.95)
Dwelling (rural)	Reference		Reference		Reference		Reference	
PA^a^ (inactive)	0.61(0.39)	1.84 (0.85–3.98)	−0.32 (0.45)	0.73 (0.30–1.77)	0.26 (0.38)	1.30(0.62–2.72)	0.29 (0.20)	1.33 (0.90–1.96)
PA^a^ (active)	Reference		Reference		Reference		Reference	
Neutral^b^								
Intercept	0.85 (0.26)		0.32 (0.25)		−0.24 (0.25)		0.68	
Age	−0.01 (0.00)	0.99 (0.98–1.00)^**^	0.00 (0.45)	1.00 (0.99–1.00)	0.01 (0.00)	1.01(1.00–1.01)^*^	−0.001 (0.00)	1.00 (0.99–1.01)
Income	0.04 (0.05)	1.04 (0.94–1.15)	0.05 (0.05)	1.00 (0.91–1.10)	−0.06 (0.05)	0.94 (0.85–1.04)	0.17 (0.07)	1.18 (1.04–1.35)
Sex (female)	−0.31 (0.10)	0.73 (0.60–0.89)^*^	−0.40 (0.10)	0.62 (0.52–0.75)^**^	− 0.39 (0.10)	0.68 (0.56–0.82)^**^	0.08 (0.13)	1.09 (0.84–1.41)
Sex (male)	Reference		Reference		Reference		Reference	
Dwelling (urban)	−0.22 (0.13)	0.80 (0.62–1.03)	−0.42 (0.13)	0.75 (0.59–0.96)	− 0.05 (0.13)	0.95 (0.74–1.22)	−0.08 (0.17)	0.92 (0.66–1.29)
Dwelling (semi-urban)	−0.21 (0.15)	0.81(0.61–1.09)	−0.25 (0.14)	0.81(0.61–1.07)	0.05 (0.15)	1.05(0.79–1.39)	−0.10 (0.20)	0.91(0.62–1.33)
Dwelling (rural)	Reference		Reference		Reference		Reference	
PA^a^ (inactive)	0.36 (0.13)	1.44 (1.12–1.83)^*^	0.15 (0.12)	1.17 (0.92–1.50)	0.29 (0.13)	1.34 (1.05–1.71)	0.50 (0.16)	1.64 (1.21–2.23)^*^
PA^a^ (active)	Reference		Reference		Reference		Reference	
*R*^2^ (Nagelkerke)	.025			.032	.034		.015	

^*^ *p* < .01, ^**^ *p* < .001

^a^Those who do (vs. do not) achieve 150 min of moderate-to-vigorous physical activity a week

^b^The reference category is: agree

## Discussion

The current study assessed the social climate of physical (in)activity in Canada and to the best of our knowledge, represents the first focused evaluation of a range of indicators that potentially reflect this construct. As an initial benchmark, interpreting the findings remains speculative until longitudinal tracking is completed to identify whether social climate changes over time. One positive finding is that Canadians perceive physical inactivity as a serious public health issue comparable to tobacco use and unhealthy diets. Perhaps reflecting the impending legalization of cannabis in Canada and fewer campaigns highlighting potential health risks [46, 47], cannabis use was rated as the least serious public health issue. In contrast, descriptive norms regarding physical activity could be considered low. This identifies room for improvement over time that may be tracked concurrent with changes in physical activity at a population level resulting from the implementation of new policies, programs or broader geopolitical events including climate change.

Complementary to using social climate as an indicator of changes at the population-level, is the future consideration of interventions targeting these social climate indicators. From the social norms perspective [21, 22], individuals may be more likely to participate in physical activity when they believe society or people important to them expect them to do so [23, 48]. There has been ongoing efforts to change social norms regarding physical activity through community-wide media campaigns (e.g., ParticipACTION’s campaigns in Canada [49], VERB in the USA [50], ACTIVE for LIFE in England [51]). Media campaigns have been proposed as a potential influencer of physical activity of the whole population by reframing the salience of social norms around the behavior into campaign messages [52]. Yet, the effectiveness of such campaigns is likely to be modest without increases in access and opportunities to be physically active. Ironically, support for media campaigns in our study was relatively weaker compared to other policy options and this may reflect broader acknowledgement of the limits of education only approaches to physical activity promotion.

The majority of participants viewed physical inactivity as a result of both personal and societal factors and that both private and public health approaches are needed to solve the inactivity problem. This indicates the importance of emerging social ecological approaches for interventions to concurrently tackle individual- as well as societal-level influencers of physical activity. This contrasts with a previous finding that the majority of Canadian policy influencers perceived physical activity to be a personal responsibility [17]. It may be that the differences are due to the target behavior – physical inactivity in the present study and physical activity in the previous study [17]. Public health policy support depends on public attribution on the problem [9, 30]. Decreases in internal attributions and increases in external attributions of physical inactivity revealed in the present study may reflect a shift in Canadians’ views on accepting societal support and policy interventions for addressing physical inactivity.

Though most policy actions to promote physical activity were well supported by the respondents, the level of support varied by the type of policy actions. Policies targeting individual responsibility for behaviors, modifying the environment including the built environment and school settings, and targeting economic levers were highly supported whereas legislative actions were much less supported. This is consistent with the previous research that public support for policy interventions generally tends to decrease as their level of “intrusiveness” or “force” increases [31]. However, such tendency can also be different by the target behavior (e.g., intrusive interventions for tobacco control being more supported than those for addressing unhealthy diet and physical inactivity) [31].

A normative approach to reducing tobacco use was a successful example of changes in public perception that tobacco use is neither mainstream nor socially acceptable, helping to reduce smoking prevalence [12]. Compared to unhealthy diet and physical inactivity, a high level of awareness exists regarding the harm from tobacco and the effects of second- and third-hand smoke; this likely led to greater public acceptance of intrusive regulations restricting tobacco use. Or, it could be that public acceptability of restrictions on smoking is increased once these restrictive policies are introduced [53, 54]. A longitudinal assessment of changes in the social climate of physical activity including different policy approaches will be needed to guide population-level physical activity interventions. However, without a similar side-effect and public health concern as environmental tobacco smoke, there may be limits to how much the social climate for physical (in)activity can be modified.

The present research is the first attempt to assess social climate using a large representative sample of Canadians and it addresses an important gap in knowledge needed for advocating for, and implementing population-level physical activity interventions. The measures utilized in the present study generally demonstrated strong test-retest reliability. One measure assessing social acceptability of physical inactivity did not. It may be speculated that the double negative nature of the statement may have caused confusion in its interpretation. In addition, it may not measure social climate but another construct –stigmatization [55] – whether people who are physically inactive are socially *devalued* and *negatively appraised* if they do not meet society’s normative expectations.

Suggesting some evidence of construct validity, the social climate indicators were associated with other demographic variables in directions that could be hypothesized. The associations of sex and age with social climate are consistent with previous research in that being female and older age were positively associated with higher support for all alcohol policy approaches [32] and obesity prevention policies [36, 56, 57]. Where people live was also differentially associated with social norms: urban and semi-urban residents were more likely to report seeing people walking, exercising, and kids being active outdoors in their neighborhood compared to those living in rural settings. Importantly, respondents’ level of physical activity participation predicted most of the social climate dimensions. People who met the Canadian physical activity guidelines perceived physical inactivity more seriously as a health problem than those who did not meet the guidelines. Active respondents perceived higher social norms of physical activity and their level of policy support was higher than inactive respondents. Our findings are consistent with other research such that public support for policies addressing health issues is associated with behavior. For example, those who exercised regularly are more likely to support obesity policy [16] and heavy drinkers were less likely than non-drinkers and ex-drinkers to be supportive of all alcohol policies, especially restrictive actions that limited their own access to alcohol [32]. Overall, the social climate of physical activity was predicted by respondents’ demographics and behavioral factors including physical activity participation.

Despite the noted strengths of the present study, it is not without limitations. Due to the cross-sectional design of the research, causal relationships cannot be inferred, and generalizability cannot be assumed. Given the self-report nature of the study, there is also the possibility of social-desirability bias. For example, given the choices of the cause of the solutions for physical inactivity, the most socially desirable choice is one where everyone is partly to blame (i.e., both individual and society), which was the most common response. In the future, using a forced choice without hybrid choices or at least a measure that presents a continuum score for respondents is recommended to attenuate social desirability bias. The physical activity assessment itself is likely a source of bias, although in comparison to other self-report tools, there is evidence of satisfactory criterion validity [44]. As the survey was administered in winter there is a possibility that the responses reflect seasonal effects even though respondents were asked to consider their answers based on spring or fall seasons. Finally, although our focus was on physical (in)activity, understanding the social climate of physical (in)activity is less clear without the simultaneous and comparative measurement of social climate for other health behaviors such as alcohol use or healthy diets. Such comparisons would be beneficial and suggest a possible need for further measurement development in comparing and contrasting the social climate of different chronic disease risk factors.

## Conclusion

The outcome of the current assessment provides a baseline for tracking the impact of future system-level interventions on social climate in Canada as it pertains to physical (in)activity. When “being active needs to be the Canadian norm, not the exception” [58] then surveillance mechanisms are needed to track progress toward this goal. Our study addresses this measurement gap and provides a snapshot of the social climate in Canada in 2018. There are some promising findings in the acknowledgement of physical inactivity as a serious public health issue and attributions for physical inactivity that go beyond individual blame. This in turn forecasts public acceptability for innovative policy intervention to address the problem. In contrast, descriptive and injunctive norms for exercise and physical activity were low. Future tracking is needed to identify any temporal (in)stability of these constructs over time and explore the likely bidirectional relationship between physical activity participation and its related social climate.

## Abbreviations

MVPA: Moderate to vigorous-intensity physical activity
PA: Physical activity
PAAQ: Physical activity for adults questionnaire
